# MK2-Dependent p38b Signalling Protects *Drosophila* Hindgut Enterocytes against JNK-Induced Apoptosis under Chronic Stress

**DOI:** 10.1371/journal.pgen.1002168

**Published:** 2011-08-04

**Authors:** Gerhard Seisenbacher, Ernst Hafen, Hugo Stocker

**Affiliations:** 1 Institute of Molecular Systems Biology, Swiss Federal Institute of Technology Zurich (ETH Zurich), Zurich, Switzerland; 2 Competence Center for Systems Physiology and Metabolic Diseases (CC-SPMD), ETH Zurich, Zurich, Switzerland; University of California San Francisco, United States of America

## Abstract

The integrity of the intestinal epithelium is crucial for the barrier function of the gut. Replenishment of the gut epithelium by intestinal stem cells contributes to gut homeostasis, but how the differentiated enterocytes are protected against stressors is less well understood. Here we use the *Drosophila* larval hindgut as a model system in which damaged enterocytes are not replaced by stem cell descendants. By performing a thorough genetic analysis, we demonstrate that a signalling complex consisting of p38b and MK2 forms a branch of SAPK signalling that is required in the larval hindgut to prevent stress-dependent damage to the enterocytes. Impaired p38b/MK2 signalling leads to apoptosis of the enterocytes and a subsequent loss of hindgut epithelial integrity, as manifested by the deterioration of the overlaying muscle layer. Damaged hindguts show increased JNK activity, and removing upstream activators of JNK suppresses the loss of hindgut homeostasis. Thus, the p38/MK2 complex ensures homeostasis of the hindgut epithelium by counteracting JNK-mediated apoptosis of the enterocytes upon chronic stress.

## Author Summary

The gut epithelium forms the first barrier against pathogens and stressors in the gut lumen, and a loss of this defence function can result in intestinal diseases. Damage in the gut epithelium triggers the proliferation of intestinal stem cells to replenish the epithelium. However, little is known about how the enterocytes are protecting themselves against stressors. We addressed the function of stress-activated protein kinase (SAPK) signal transduction pathways in the larval gut of *Drosophila*. Our study revealed that a particular module of the p38 SAPK signal cascade is required to protect the larval hindgut epithelium against chronic salt stress. We identified the two kinases, p38b and MK2, as key components of this protective signal. In the absence of p38b or MK2, the stress-inducible JNK cascade is locally upregulated and eventually induces apoptosis. Although the function of the p38b/MK2 module is only required in the enterocytes, the elimination of the affected cells results in atrophy of the overlaying muscle layer and subsequent systemic defects in the larvae (e.g., induction of antimicrobial peptides). We hope that our findings will contribute to a better understanding of early (i.e., pre-inflammatory) events in the development of human intestinal diseases.

## Introduction

In its function as a protective barrier, the intestinal epithelium is constantly exposed to stressors from the outside [1]. It acts as a mediator between the bacterial flora and the host's immune system, and the intestinal epithelial cells have to respond to extrinsic and intrinsic factors to ensure their own survival and proper gut homeostasis. Two populations of cells have to orchestrate different aspects of intestinal epithelium survival. While the intestinal stem cells (ISCs) are essential for the proliferative aspects of intestinal homeostasis [2]–[4], the enterocytes (ECs) form the first line of defence against pathogens and stressors. Signalling cascades that are modulated by external signals and by cellular stress are crucial regulators of intestinal epithelial survival. For example, mice with gut-specific knockout of *NEMO* spontaneously develop intestinal lesions reminiscent of those in inflammatory bowel diseases (IBDs), indicating an essential role for NFκB signalling in EC survival [5], [6]. Recently, ER stress in the ECs has also been found to influence epithelial homeostasis, and mutations in *XBP1* are sufficient to trigger an IBD-like phenotype [7]. The p38 stress-activated protein kinase pathway has also been implicated in intestinal disorders [8] but its role in intestinal diseases is still controversially discussed [9], [10].

The p38 SAPK belongs to the MAPK family and is conserved from yeast to humans. In higher eukaryotes, p38 associates with its major target, the MAPK-activated protein kinase MK2. This complex resides in the nucleus in the resting state. Upon stress, p38 is activated by MKK3/MKK6 and phosphorylates MK2, which results in an exposure of the nuclear export signal of MK2 and a subsequent nuclear export of the complex [11]. Another consequence of the p38/MK2 complex formation is the stabilisation of p38 protein. Interestingly, the kinase activity of MK2 is required neither for the nucleo-cytoplasmic shuttling nor for the p38 protein stabilisation [11]–[13]. Conversely, MK2 kinase activity is crucial to phosphorylate small heat-shock proteins, transcription factors (e.g., SRF and HSF-1), and TTP [14]–[16]. The inhibitory phosphorylation of TTP by the p38/MK2 complex has been shown to increase the translation of AU-rich elements (ARE)-containing mRNAs including the mRNA encoding the proinflammatory cytokine TNFα [17]. Furthermore, p38 and MK2 have been shown to act as cytoplasmic checkpoint kinases in parallel to CHK1 [18], [19]. Due to the plethora of p38/MK2 functions, targeting the p38 SAPK branch with inhibitors might lead to harmful side effects. Thus, it is important to understand the roles of p38 signalling in a tissue-specific context. The availability of mouse models has helped to decipher some *in vivo* roles of p38 SAPK signalling [20], [21] but the complex nature of the intestinal system has hampered a detailed analysis.

Studies in the model organism *Drosophila* have provided new insights into intestinal maintenance and how different signalling pathways are employed to ensure proper gut homeostasis. The *Drosophila* gut consists of the fore-, the mid-, and the hindgut. The larval and adult midgut displays a regional specification along the antero-posterior axis and fulfils vital functions such as nutrient absorption [22]. The main part of the embryonic and larval hindgut, the large intestine, is subdivided into a ventral (hv, positive for *Delta* expression) and a dorsal domain (hd, positive for *engrailed* expression) [23] that are separated by a single row of boundary cells [24]. The adult hindgut shows a similar yet more complex organisation [25]. The function of the hindgut, however, remains largely unknown. Ultrastructurally, the hd domain is marked by deep infoldings and enriched in elongated mitochondria, resembling the rectal papillae of other insects. Based on these similarities, it has been speculated that the *Drosophila* large intestine plays a role in ion and/or water resorption [23], [24].

At least four distinct intestinal epithelial cell types are found in *Drosophila*: intestinal stem cells (ISCs), the hormone-producing enteroendocrine cells (EEs), enterocytes (ECs) and the transient enteroblasts (EBs; progenitors of EEs and ECs). In the adult midgut, ISCs are required for normal gut homeostasis, but in aged and/or stressed individuals the number of midgut ISCs is increased and differentiation is disturbed [26], [27]. The orchestrated activation of Hippo, JNK, JAK/STAT, Notch and EGFR signalling within and between the ISCs, the ECs and the visceral muscles is required to coordinate proliferation, differentiation and cellular turnover in the midgut epithelium [26], [28]–[36]. In contrast to the situation in the midgut, the hindgut stem cells are not required for hindgut homeostasis in the larva and in the adult fly. The hindgut ISCs are rather needed during the shift from larval to adult hindgut, and stress induces proliferation and cell migration in the pylorus region [37]. Thus, especially the adult midgut serves as a good experimental model for the analysis of EC replenishment upon damage, but the mechanisms governing proper stress response in the ECs have remained elusive.

p38 SAPK signalling also plays a role in gut homeostasis in *Drosophila*. In aged adult midguts, an increase in p38b expression has been observed in *Delta*-positive stem cells, which appears to be partly due to DREF-mediated transcriptional activation [38], [39]. Knockdown of p38b in the ISCs prevents age- and stress-induced ISC overproliferation and accumulation of aberrant *Delta*-positive cells, implying a role for p38b in regulating intestinal regeneration [38]. p38 signalling has been shown to be required for DUOX expression in differentiated ECs and for normal differentiation in the ISCs [38], [40]. Consistently, a recent study showed that the larval intestinal epithelium is more susceptible to damage by pathogens in the absence of p38 function [41]. However, the mechanism of p38 action within the ECs remains unclear. In *Drosophila*, p38 signalling can antagonize the closely related JNK SAPK branch [42]. JNK signalling has also been shown to regulate several aspects of intestinal function. It is required in the midgut ECs to induce autophagy and thereby ensure their survival during oxidative stress [43]. In the ISCs, JNK is needed for proper stress response but strong activation of JNK leads to differentiation defects and loss of gut homeostasis [26]. Whether p38 and JNK influence each other in their intestinal function has not been addressed so far.

In this study, we investigate how the *Drosophila* larval hindgut is enabled to maintain homeostasis under stress conditions. Using deletion mutants for *MK2*, *p38a* and *p38b*, we show that MK2 and p38b form a complex that is specifically required to protect the dorsal hindgut ECs against chronic stress. In the absence of this p38b/MK2 complex, JNK is activated in patches of hindgut ECs, resulting in JNK-dependent apoptosis, loss of epithelial organisation, and melanisation of hindgut regions. This melanisation of the ECs does not require the recruitment of hemocytes, indicating an epithelial response that might also precede immune activation in mammalian intestinal diseases. Thus, we identify a specific SAPK signalling module required to maintain hindgut epithelial integrity upon stress.

## Results

### *Drosophila MK2* Is Dispensable for Normal Development and Survival at Non-Stress Conditions

**Figure 1:**
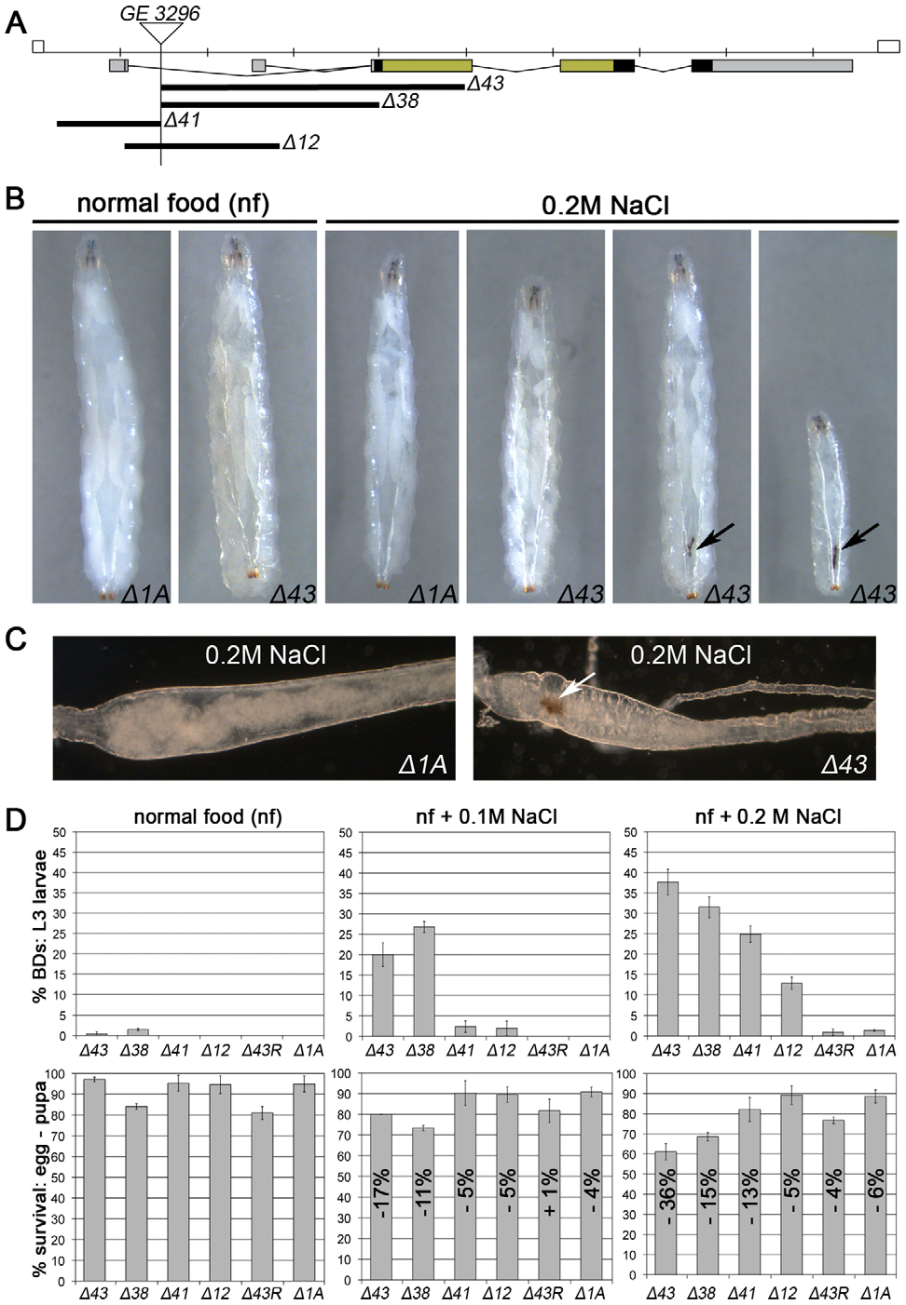
*MK2* deficient larvae are sensitive to salt stress. (A) Deletion alleles of *MK2* generated by imprecise excision of the P-element GE3296. The untranslated regions (UTRs; grey), protein coding sequence (black), and the sequences encoding the kinase domain (green) are indicated. (B) *MK2* null mutants (*Δ43*) but not wild-type larvae (*Δ1A*) develop melanisations in the posterior part of the body (black dots/BDs, indicated by arrows) when reared on 0.2 M NaCl food. (C) Bright field pictures of wild-type (*Δ1A*) and *MK2* mutant (*Δ43*) hindguts of larvae reared on 0.2 M NaCl food. Rectum points to the left; BD indicated by arrow. (D) The different *MK2* deletion alleles reared on normal food, 0.1 M and 0.2 M NaCl food, respectively, reveal an increase in BDs with the severity of the stress (upper panel) and a reduction in survival (lower panel) as compared to the control (*Δ1A*). The decrease in the survival rate is indicated relative to the survival on normal food of the respective genotype. A genomic *MK2* rescue construct fully rescues both the BD phenotype and the decreased survival of *MK2* mutants (*Δ43R*).

The mammalian MAPKAP-K2 is known to be a downstream kinase of the p38 branch of SAPKs [44]. To generate deletion mutants for *Drosophila MK2*, we mobilised a P-element insertion located in the *MK2* locus (Figure 1A). Whereas *Δ43* is a null allele as judged by the absence of MK2 protein and by the failure of *Δ43* larval lysates to phosphorylate mammalian small heat shock protein 25 in a kinase assay (Figure S1A), the alleles *Δ41* and *Δ12* are likely to represent hypomorphic alleles. *Δ38* is probably also a null allele although the generation of a truncated protein (initiated from alternative Methionine codons) cannot be excluded (Figure 1A). A precise excision allele of the same P-element (*Δ1A*) was used as control throughout this study. All of the generated alleles show no obvious phenotypic alteration and can be kept as homozygous lines at normal conditions. Since several mutants of the SAPK pathway are sensitive to stresses including high osmolarity [45], [46], we tested the *MK2* mutants in various stress assays including oxidative stress (paraquat feeding), UV exposure during late embryonic/early larval development, heavy metals (0.5 mM copper sulphate), high osmolarity (0.2 M NaCl) and SDS (0.2%) (Figure S1B and data not shown). Interestingly, only salt and SDS feeding resulted in a melanotic phenotype in 35% to 45% of the mutant larvae, characterized by the appearance of a “black dot” (BD) in the posterior part of the body (Figure 1B and Figure S1C–S1E). Closer examination revealed that the BD localised to the posterior hindgut, and the affected hindgut epithelium appeared pathologically altered (Figure 1C). All *MK2* alleles were analysed for the appearance of BDs and survival at three conditions: normal food, weak salt stress (0.1 M NaCl) and strong salt stress (0.2 M NaCl) (Figure 1D). The behaviour of *MK2* hypomorphic larvae indicated that the levels of MK2 get more important with increasing osmolarity. Introducing a genomic rescue construct completely rescued both the BD phenotype and the lethality of *Δ43* mutants at 0.2 M NaCl (Figure 1D). Thus, MK2 is not essential at normal conditions but is required when larvae are reared on a high sodium chloride diet.

### *Drosophila MK2* Is Required to Protect the Hindgut from Stress-Induced Apoptosis

**Figure 2:**
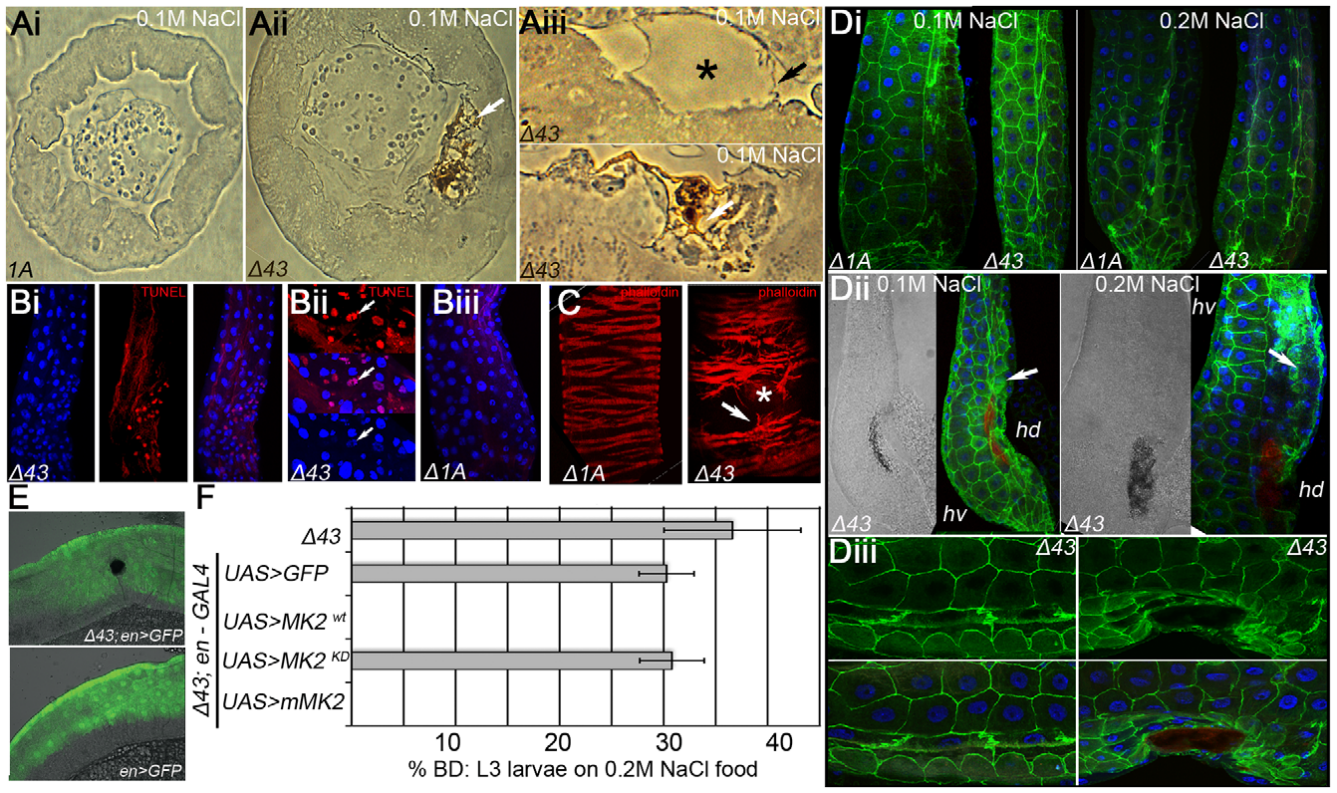
Increased apoptosis and tissue damage in hindguts of *MK2* mutants. (A) *MK2* mutants show ruptures of ECs when reared on high salt diet. Bright field pictures of sections of hindgut tissue of larvae reared on 0.1 M NaCl food are shown. (Ai) wild-type (*Δ1A*), (Aii) *MK2* mutant (*Δ43*) with BD (white arrow). (Aiii) Higher magnifications of ECs in the dorsal hd domain of *MK2* mutants. Blistering of the apical part (upper panel, black asterisk) and melanisation of the apical region (lower panel, white arrow) are frequently observed. The apical surface of the ECs appears to be intact (black arrow). (B) Apoptosis is increased in the hindguts of *MK2* mutant larvae reared on high salt conditions. (Bi) TUNEL staining revealed local apoptosis in *MK2* mutant hindguts. (Bii) Magnification of apoptotic foci shows the condensed nature of the TUNEL positive nuclei (white arrows). (Biii) No apoptotic cells are present in control hindguts. (C) *MK2* mutant larvae reared on high salt food (0.2 M) with large BDs show ruptures of the visceral muscles (right panel, white arrow) in comparison to the regular organisation of wild-type larvae (left panel). The location of the BD is marked by an asterisk. (D) The cell-cell junction marker Nrg-GFP indicates morphological defects in ECs in the hd domain. (Di) Wild-type and *MK2* mutant larvae reared on 0.1 M or 0.2 M NaCl food show normal localisation of Nrg-GFP in the ECs. The hd domain points to the right; the rectum points to the bottom. (Dii) In *MK2* mutants with BDs, Nrg-GFP is more diffusely localised at the sites of BDs (white arrow). The BD is discernible in the bright field picture and by its autofluorescence in the far-red channel (red) (Diii) Higher magnification of Nrg-GFP (green) signal in *MK2* mutants (reared on 0.2 M NaCl food) with (right) or without BD (left). (E) BDs are always found in the *engrailed-*positive hd domain (green) of the hindgut. (F) Rescue of the *MK2* mutant phenotype by *en-GAL4* mediated expression of the indicated cDNA constructs.

We speculated that MK2 is specifically required to protect the hindgut epithelium. In sections of the hindgut, dorsal ECs with BDs were apically ruptured (Figure 2Aii). In wild-type hindguts, ECs of the dorsal domain were not damaged under salt stress conditions (Figure 2Ai and Figure S2C). Similarly, most *MK2* mutant hindguts without BDs did not display morphological alterations (Figure S2C). However, a blistering of the apical surface without damaging the apical membrane was occasionally observed (Figure 2Aiii and Figure S2C). Blistering of the apical surface was also observed in *MK2* mutant hindguts with BDs in regions away from the BD (Figure S2C). The melanisations occurred at the apical surface within the ECs, probably preceded by the blistering (Figure 2Aiii and Figure S2C). Next, we tested the hindgut tissue for the presence of dying cells. TUNEL staining revealed local clusters of apoptotic hindgut ECs in *MK2* mutant larvae (Figure 2Bi and 2Bii) but not in wild-type larvae reared on 0.2 M NaCl (Figure 2Biii). The visceral muscles surrounding the hindgut ECs appeared unaffected in *MK2* mutant larvae. A disturbed hindgut musculature was only observed in hindguts with large BDs (Figure 2C). Furthermore, loss of epithelial integrity was evident from the mislocalisation of Neuroglian (Nrg) (Figure 2D). Nrg-GFP was localised laterally in cell-cell junctions in hindguts of wild-type and *MK2* mutant larvae (Figure 2Di). In contrast, in *MK2* mutant larvae with BDs, Nrg-GFP was normally localised in unaffected regions but displayed a more diffuse pattern close to the BD (Figure 2Dii). This mislocalisation was more pronounced at 0.2 M NaCl (Figure 2Dii and 2Diii). Interestingly, BDs were only found in the dorsal hindgut (hd) compartment, as judged by cell and nuclear size (hd is composed of smaller cells) (Figure 2Dii and 2Diii).

We performed a series of rescue experiments to determine where MK2 function is required. The BD phenotype was rescued by ubiquitous and hindgut-specific but not by midgut-specific expression of *MK2* (Figure S2D). Moreover, only wild-type MK2 but not a kinase-dead version of MK2 rescued the BD phenotype. The largest domains of the larval hindgut are the dorsal *engrailed*-positive (hd) and the ventral *Delta*-positive (hv) domain. BDs were only observed in the hd domain (Figure 2E). Consistently, *engrailed-GAL4* driven expression of a wild-type *Drosophila* MK2 or a wild-type murine MK2, but not of a kinase-dead *Drosophila* MK2, rescued the BD phenotype (Figure 2F).

Melanisation in insects can be regarded as a wound healing or defence response and has been shown to be either hemocyte-dependent or hemocyte-independent [47]. We thus attempted to clarify whether BD formation in *MK2* mutant larvae was dependent on the recruitment of hemocytes. Staining for the blood cell marker Hemese - either direct (using antibodies) or indirect (using *He-GAL4 UAS-GFP*) - revealed the absence of blood cells at or within the hindgut epithelium in both wild-type and *MK2* mutant hindguts (Figure S2A and data not shown). Consistently, the formation of BDs was still observed in hemocyte-ablated *MK2* mutants (Figure S2B).

Together, those results show that *Drosophila* MK2 kinase function is required in the dorsal hindgut compartment to protect larval hindgut ECs from stress-induced apoptosis upon salt stress.

### *MK2* Genetically Interacts with *p38a* and *p38b*

**Figure 3:**
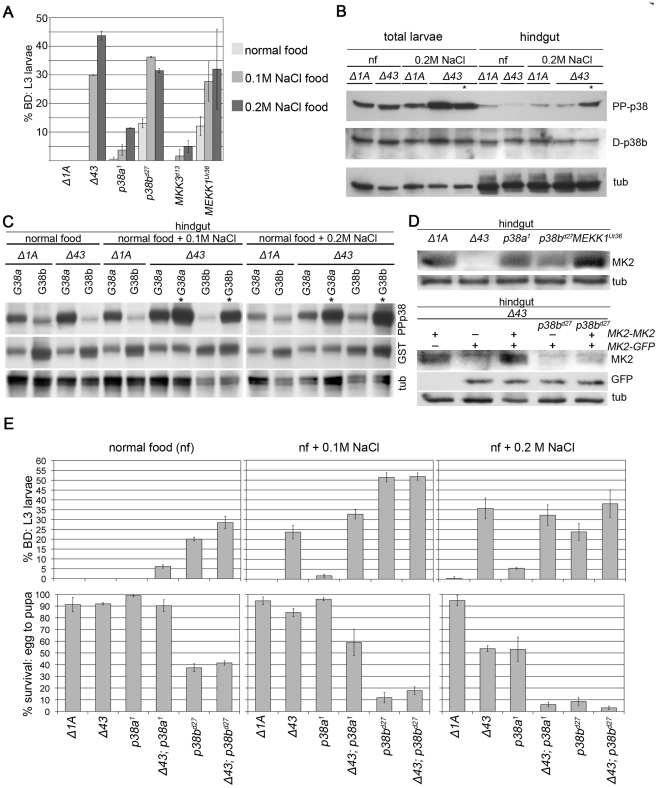
Interactions of MK2 with the p38 SAPK pathway. (A) Mutations in genes encoding p38 SAPK pathway members were tested for a BD phenotype (normal food, 0.1 M NaCl, 0.2 M NaCl). *MEKK1* and *p38b* but not *MKK3* or *p38a* show a BD phenotype similar to *MK2* mutants. The wild-type control (*Δ1A*) does not display any BDs. (B) The activating pTGpY phosphorylation (PP-p38) of p38 is increased in *MK2* mutants under stress, especially in larvae with BDs (lanes marked by asterisks). (C) GST-tagged p38a (G38a) and p38b (G38b) were overexpressed in wild-type or *MK2* mutant larvae to distinguish between the activation of the two p38 kinases. The lanes with lysates of *MK2* mutant larvae with BDs are marked by asterisks. (D) Stabilisation of MK2 happens at the level of protein stability or translation and not at the level of transcription. In *p38b* mutant hindguts MK2 protein levels are reduced (upper blot). GFP expressed under the control of the genomic *MK2* locus (*MK2-GFP*, *MK2* coding sequence replaced by sequence encoding *GFP*) is unaltered in a *p38b* mutant background. In contrast, MK2 expressed from the same genomic region (*MK2-MK2*, *MK2* coding sequence not replaced) does not result in restoration of MK2 protein levels (lower blot). (E) *p38a* and *p38b* deletion alleles (in wild-type and *MK2* mutant background, respectively) were assessed for the appearance of BDs (upper panel) and survival (lower panel) on normal food, 0.1 M NaCl and 0.2 M NaCl, respectively.

Mutations in *MEKK1* and *p38b* but not in *MKK3/lic* and *p38a* resulted in a strong BD phenotype even at normal food conditions (Figure 3A). To define the roles of p38a and p38b with respect to MK2, we next tested these kinases genetically for the behaviour at normal conditions and under salt stress (Figure 3E). At unstressed conditions, *MK2* was required neither for hindgut homeostasis nor for survival. Similarly, *p38a* mutants did not display BDs or elevated mortality rates. In contrast, *MK2; p38a* double mutants developed a weak BD phenotype, indicating that p38a and MK2 act in two parallel stress-signalling pathways. *p38b* mutants had a decreased survival rate, consistent with published findings [46]. p38b is required for hindgut homeostasis even under normal conditions, because larvae lacking p38b function developed BDs on normal food. Interestingly, *MK2; p38b* double mutants displayed a slight increase in BDs but no increase in lethality rate compared to *p38b* single mutants. Thus, *p38b* and *MK2* are likely to function in the same pathway but both kinases may have additional independent functions in the hindgut or in other tissues.

Consistent with a common MK2/p38b pathway, *p38b* single mutants and *MK2; p38b* double mutants displayed the same phenotype at both weak (0.1 M NaCl) and strong (0.2 M NaCl) stress conditions. At weak stress conditions, *p38a* was not crucial for survival and hindgut homeostasis. In contrast, *MK2* mutants displayed hindgut defects but no increase in mortality. In agreement with MK2 and p38a acting in two parallel stress-signalling pathways, BD formation was only slightly increased in the *MK2; p38a* double mutants but the absence of *MK2* significantly enhanced the mortality of *p38a* mutants. This lethality increase of *MK2; p38a* double mutants was also observed at strong salt stress. At this condition, both *MK2* and *p38a* mutants resulted in increased mortality rates, indicating that both branches of stress signalling are required for survival. Whereas MK2 is specifically required in the hindgut, p38a might be needed in other organs since *p38a* mutants hardly developed BDs.

Taken together, our genetic data suggest the existence of two major p38 branches in *Drosophila* that are required to varying extents during normal and different salt stress conditions. The *p38a* branch is not essential at normal or weak salt stress conditions but required at strong salt stress conditions. In contrast, the *p38b* branch is required at both normal and salt stress conditions to protect the hindgut. Moreover, the lethality of *p38a; p38b* double mutants [46] suggests that, besides their specific functions, both p38 kinases engage in a common essential function. MK2 is involved in a sub-branch of the p38b branch and appears to be a key effector of p38b in the hindgut. MK2 becomes more important with increasing stress conditions specifically in this tissue.

### p38b Phosphorylation Is Increased in Melanised Larval Hindguts

We next checked the activation status of p38 by Western analysis (Figure 3B). Chronic exposure (from L1 to L3) to 0.2 M NaCl did not increase p38 phosphorylation in total and hindgut lysates of wild-type larvae. In contrast, stronger p38 activation was observed in total larval lysates of *MK2* mutants under stress conditions. This strong activation was also apparent in the hindgut but only in larvae with BDs. Since we were not able to distinguish the endogenous p38a and p38b, we overexpressed GST-tagged versions of p38 and analysed their activation status in the hindgut (Figure 3C). GST-p38a was strongly activated even under normal conditions, and the activation was slightly increased under stress conditions. In *MK2* mutants, GST-p38a was more strongly activated under all conditions and especially in hindguts with BDs. In contrast, GST-p38b was only weakly phosphorylated in wild-type and *MK2* mutant hindguts at all conditions, except in hindguts of *MK2* mutant larvae with BDs where a boost in GST-p38b phosphorylation was seen. Thus, the increase in endogenous p38 phosphorylation observed in *MK2* mutant larval hindguts is probably due to p38b phosphorylation, suggesting that a negative feedback loop operates from MK2 to the upstream signalling components. Alternatively, the lack of a functional stress-protective MK2/p38b function may increase the stress in the ECs and lead to a vicious cycle that boosts p38b activation.

### MK2 Protein Abundance Depends on p38b

In mammalian cells, MK2 is needed to stabilise p38 protein levels [13], which does not appear to be the case in *Drosophila* (Figure 3B and 3C). We wondered whether the protein levels of MK2 would depend on the presence of the upstream components. No change in MK2 expression was observed in *p38a* mutants, and a slight increase was detected in *MEKK1* mutants. In sharp contrast, MK2 protein levels were reduced in *p38b* mutants (Figure 3D). Using a genomic *MK2* rescue construct and a genomic *MK2*-*GFP* reporter in an *MK2* null mutant background, we found that transcription from the *MK2* locus was unchanged but protein levels were reduced, probably due to destabilisation of MK2 in the absence of p38b (Figure 3D).

### MK2 Physically Interacts with p38b

**Figure 4:**
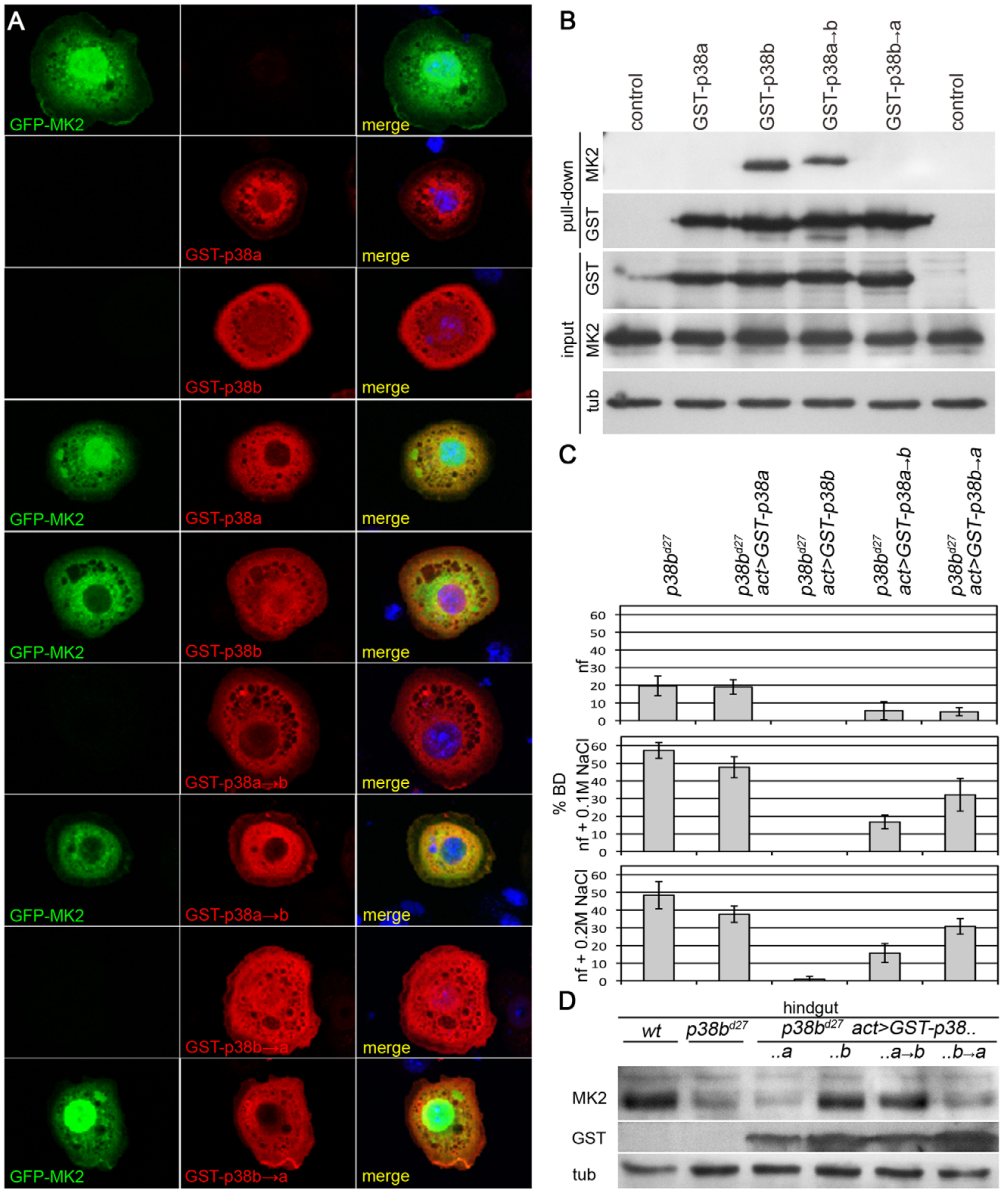
A p38b/MK2 complex is required for hindgut EC stress protection. (A) GFP-MK2 (green) localises to the nucleus. GST-p38a and GST-p38b show a broad, more cytoplasmic distribution (red). GST-p38b co-expression shuttles GFP-MK2 to the cytoplasm in a DPTD-motif dependent manner. GST-p38a or GST-p38b→a (DPTD changed to EPSV) do not change the localisation of MK2, but a GST-p38a→b protein mutant can export GFP-MK2 from the nucleus. (B) In pull down experiments, GST-p38b co-precipitates MK2, whereas p38a and the docking mutant p38b→a do not bind MK2. The DPTD motif introduced into p38 (p38a→b) is sufficient for binding to MK2. (C) Whereas overexpression of GST-p38b rescues completely, GST-p38a fails to rescue the BD phenotype of *p38b* mutants. GST-p38a→b and GST-p38b→a partially rescue the BD phenotype with p38a→b performing better. (D) Docking of MK2 to p38 that harbours a DPTD motif is sufficient to restore wild-type levels of MK2 protein in a *p38b* mutant background.

The facts that *MK2* and *p38b* genetically interact and that MK2 protein levels depend on the presence of p38b suggest a close physical interaction of the two kinases. Therefore, we expressed tagged versions of p38a, p38b and MK2 in S2 cells and checked for co-localization of these kinases. In mammalian cells, MK2 is nuclear under normal conditions and translocates to the cytoplasm upon stress [48]. Similarly, in *Drosophila* S2 cells, GFP-MK2 was mainly found in the nucleus, whereas overexpressed p38a and p38b were largely cytoplasmic (Figure 4A). Co-overexpression of p38b and MK2 (but not of p38a and MK2) resulted in a nuclear-to-cytoplasmic translocation of GFP-tagged MK2 (Figure 4A). Consistently, MK2 was found to bind p38b but not p38a in pull down experiments (Figure 4B). This interaction as well as the nuclear-to-cytoplasmic shuttling was dependent on a four amino acid motif (DPTD) in p38b resembling the common docking motif (CD domain) that is critical for docking interactions in MAPKs [49]. In p38a, the respective four amino acids are EPSV. Exchanging these motifs revealed that the DPTD motif is necessary and sufficient to dock MK2 to p38 proteins (Figure 4A and 4B).

The expression of GST-p38b completely rescued the BD phenotype of *p38b* deficient flies, whereas expression of GST-p38a had no influence on the BD phenotype (Figure 4C and Figure S3). Expressing either GST-p38a→b (p38a with docking motif of p38b) or GST-p38b→a (p38b with respective sequences of p38a) resulted in a partial rescue of the BD phenotype. On normal food, both versions rescued partially, indicating that although not essential, binding to MK2 is required for a complete rescue under normal conditions. On 0.1 M and 0.2 M NaCl, GST-p38a→b resulted in substantial but incomplete rescue, suggesting that other aspects of p38b function not mediated by binding to MK2 are required for a complete rescue. Consistently, a GST-p38b→a protein that is not able to bind MK2 also partially rescued the p38b phenotype but to a lesser extent than p38a→b (Figure 4C and Figure S3). MK2 protein levels in the hindgut were restored by expressing p38b or p38a→b but not by p38a or p38b→a (Figure 4D). Thus, binding of p38 to MK2 is required to localise and stabilise MK2 and is important for the stress-protective function in the hindgut.

### The Catalytic Activity of p38b Is Required to Localise MK2

**Figure 5:**
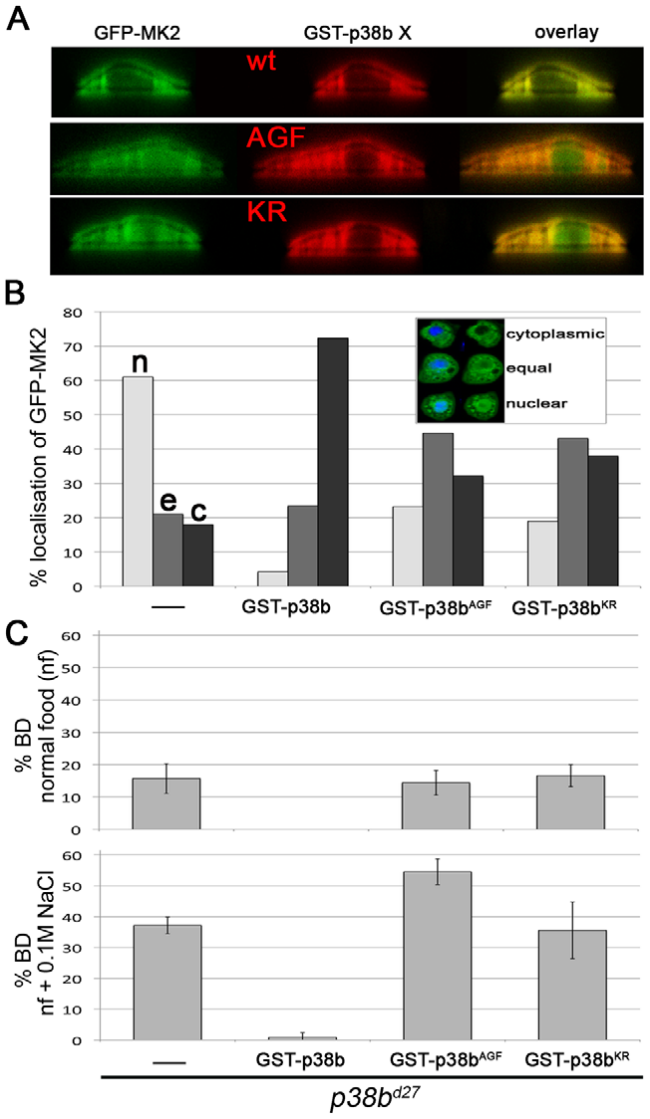
p38b activation and kinase activity are required to protect the hindgut. (A) Co-expression of GST-p38b and MK2 results in cytoplasmic localisation of GFP-MK2. When a kinase-dead (KR) or non-activatable (AGF) version of p38b was expressed, the nuclear export was not efficient. (B) Quantification of GFP-MK2 localisation upon GST-p38b, GST-p38b^AGF^ or GST-p38b^KR^ co-expression. Localisation was assigned to three classes: cytoplasmic (c), equal distribution between nucleus and cytoplasm (e), and nuclear localisation (n). (C) Activation and kinase function are required for proper function of p38b in the hindgut. The rate of BDs of *p38b* mutants upon re-expression of p38b protein variants by means of *Act-GAL4* was assessed.

The catalytic activity of MK2 and MK2 binding to p38b are required to protect the hindgut epithelial cells upon salt stress. To address whether the catalytic activity of p38b is also necessary, we used GST-tagged non-activatable p38b^AGF^ and kinase-dead p38b^KR^ protein mutants. Whereas co-expression of wild-type GST-p38b and GFP-MK2 led to a nuclear export (>70%) of MK2, the localisation of GFP-MK2 was random when GST-p38b^AGF^ or GST-p38b^KR^ were co-expressed (Figure 5A and 5B). Consistently, the BD phenotype of *p38b* null mutants was not rescued by re-expression of the kinase-dead or of the non-activatable p38b protein version (Figure 5C). Moreover, at high NaCl stress (0.2 M NaCl), the p38b^AGF^ and p38b^KR^ expressing larvae died. This could be explained by the titration of an upstream kinase of p38b, which might impinge on the activation of other downstream effectors. Thus, the catalytic activity of p38b is required to impact on the subcellular localisation and thereby the proper function of MK2.

### JNK Signalling Is Induced in *MK2* Mutants and Is Linked to Apoptosis

**Figure 6:**
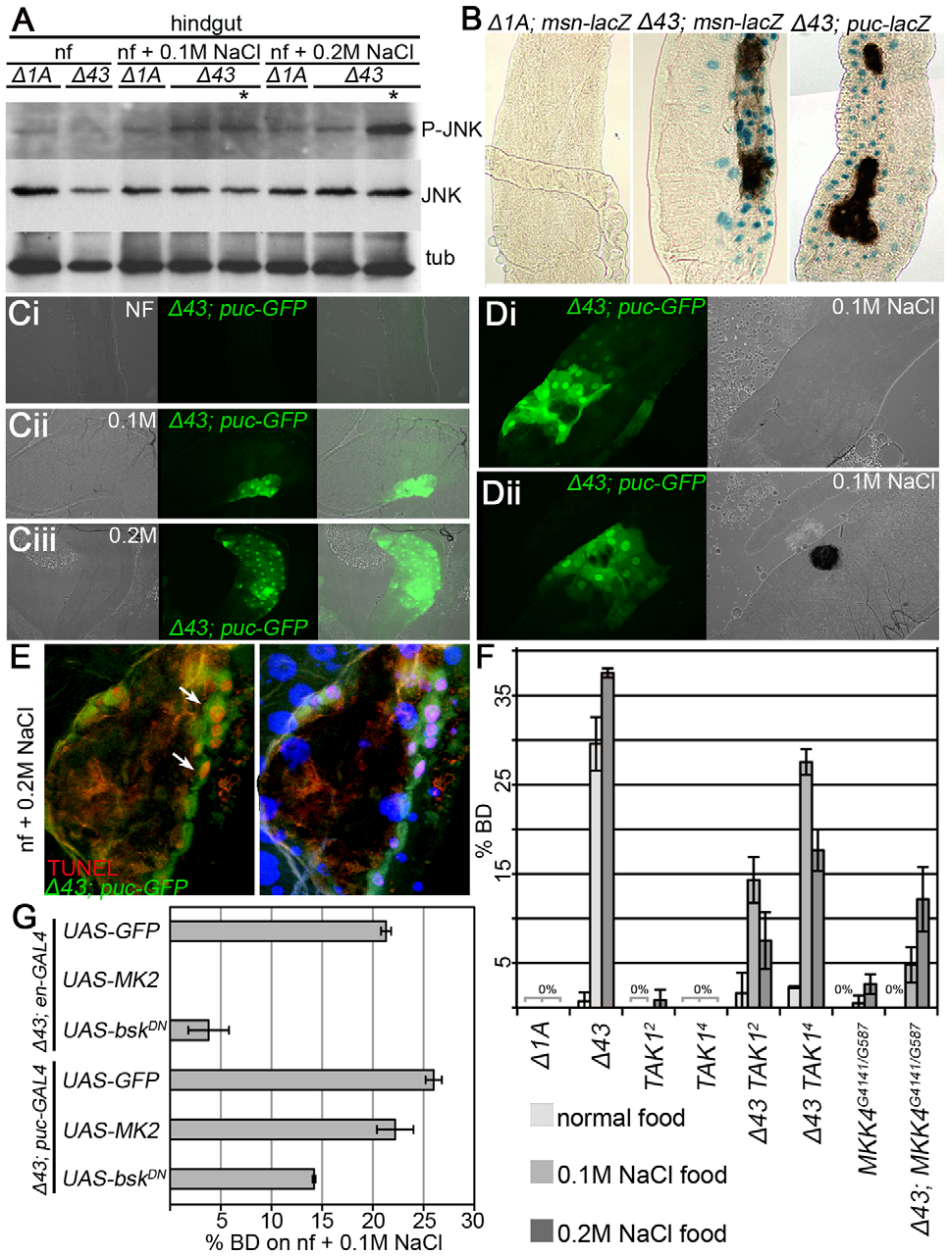
JNK activation leads to EC apoptosis in *MK2* deficient hindguts. (A) JNK phosphorylation is strongly increased in hindguts of *MK2* mutant larvae with BDs reared on 0.2 M NaCl food. Lanes containing lysates of larvae with BDs are marked by asterisks. (B) Reporters of JNK signalling activity (*msn-lacZ* and *puc-lacZ*) in wild-type (*Δ1A*) and *MK2* mutant (*Δ43*) larvae reared on 0.2 M NaCl. In *MK2* mutants, the JNK activity reporters get induced, especially close to the BDs. (C) Activation of *puc-GFP* in *Δ43* mutants prior to BD appearance. Whereas no activation of *puc-GFP* occurs on normal food (Ci), fields of increased *puc-GFP* appear in the hindgut epithelium on 0.1 M NaCl (Cii) and 0.2 M NaCl (Ciii). The area of the patches and the number of affected ECs correlate with the strength of the stress. (D) *puc-GFP* induction occurs prior to the melanisation process. Hindgut of an *MK2* mutant larva at the initiation of a BD (Di) and of an *MK2* mutant larva after BD appearance (Dii), both reared on 0.1 M NaCl food. (E) In *Δ43* mutants on 0.2 M NaCl, highest *puc-GFP* (green) and TUNEL staining (red) co-localise in the BD border region (white arrows). (F) Removing two copies of *TAK1* or of *MKK4* partially suppresses the BD phenotype of *MK2* mutants. (G) Overexpression of MK2 and of Bsk^DN^ in the dorsal hindgut (by means of *en-GAL4*) rescues the BD phenotype of *MK2* mutants on 0.1 M NaCl food. Whereas re-expression of MK2 using *puc-GAL4* does not rescue the BD phenotype on 0.1 M NaCl food, downregulation of JNK signalling using *UAS-bsk^DN^* in combination with the *puc-GAL4* driver results in a substantial suppression of the BD phenotype.

JNK signalling has been implicated in triggering apoptosis [50]. Furthermore, a JNK antagonizing activity of p38 signalling has been observed in developmental processes [42] and at the systemic level during infection [41]. Thus, we tested whether the cell death observed in *MK2* mutant larvae reared on high salt correlated with JNK activation. Indeed, elevated levels of phosphorylated JNK were detected in hindgut lysates of *MK2* mutant larvae with BDs (Figure 6A). As *in vivo* readouts for JNK signalling activity, we used reporters for *misshapen* and *puckered* [51], [52]. An induction of both *msn>lacZ* and *puc>lacZ* was observed in *MK2* mutant larval hindguts, with highest levels adjacent to the BDs (Figure 6B). To exclude that the induction of JNK signalling is a secondary consequence of wound healing or the melanisation process, we checked for *puc-GFP* induction in stress-challenged *MK2* mutant larvae before BD formation. Interestingly, patches with *puc-GFP* signal were readily detected upon stress in larvae devoid of BDs, and the area of those patches correlated with the strength of the stress (Figure 6C). We noted that *puc-GFP* was activated in a graded fashion, with highest activity where ECs undergo apoptosis and a BD will ultimately form (Figure 6D). Strong *puc-GFP* reporter activity co-localised with TUNEL positive cells close to the BDs (Figure 6E). Removing the JNK upstream components *TAK1* and *MKK4*, respectively, in an *MK2* mutant background partially suppressed the BD phenotype, indicating that the hindgut epithelial cells are dying due to JNK-induced apoptosis (Figure 6F). Expressing a dominant-negative version of JNK (Bsk^DN^) in the dorsal domain using *engrailed-GAL4* resulted in a suppression of the BD phenotype (Figure 6G). Furthermore, expression of Bsk^DN^ specifically in cells with high JNK activity (using *puc-GAL4*) substantially suppressed BD formation. In contrast, re-expression of MK2 at this stage reduced the number of larvae with BDs only mildly (Figure 6G). Thus, deregulated JNK activation in the hindgut of *MK2* mutants precedes and probably causes cell death and BD formation.

## Discussion

Gut homeostasis—under normal and stress conditions—is ensured by complex interactions between the intestinal epithelium, the immune system and the gut flora. *Drosophila* has been used as a simple model organism to address different aspects of intestinal homeostasis. Replenishment of the gut epithelium by ISCs clearly contributes to epithelial homeostasis but how the differentiated ECs are protected against stressors has remained largely unknown. We used the larval hindgut of *Drosophila* as a simple intestinal model organ to address how stress signalling in the hindgut ECs ensures intestinal homeostasis in the absence of proliferative cells. Our analysis identifies the p38b/MK2 signalling module as a critical component in the protection of hindgut ECs against salt stress.

**Figure 7:**
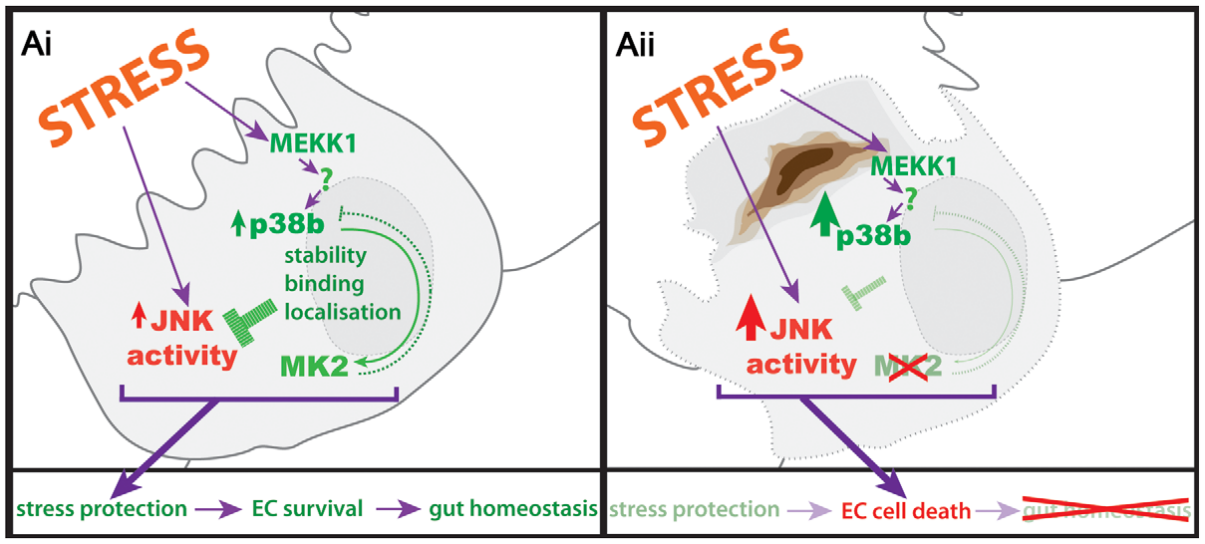
Protection of hindgut ECs by p38b/MK2. (Ai) Stressors in the gut lumen act on the hindgut ECs and induce p38 and JNK activation. The interaction of p38b with MK2 influences the localisation and protein stability of MK2, ensuring proper stress response by keeping JNK activity low. (Aii) In the absence of a functional p38b/MK2 complex, stress protection of the ECs is reduced and JNK activity is no longer kept in check, resulting in EC cell death and loss of hindgut homeostasis.

We propose a model that puts a p38b/MK2 complex in the centre of stress-protection of the hindgut ECs (Figure 7). In the absence of this signalling module, cells are undergoing JNK-dependent apoptosis upon stress. The lesion in the EC monolayer results in the damage of the overlying hindgut musculature. This regional loss of the barrier function leads to systemic defects in the larvae (Figure S4), further weakening the larvae and impairing growth under stress conditions. As a consequence, pathogens and toxic substances might enter the body cavity, eventually resulting in the melanisation of pericardial cells and the induction of cecropin in the midgut (Figure S4).

Interestingly, JNK activation in *MK2* mutant hindguts precedes the melanisation, and it consistently occurs in patches. Within these areas, some cells acquire highest amounts of JNK activity and eventually undergo apoptosis. The surrounding cells maintain JNK activity, forming a rim around the scar in the tissue. The number of affected ECs remains roughly constant for a given stress. Presently we do not know what determines the patches with high JNK activity within the tissue. Although the hd domain ECs of the hindgut form a homogeneous epithelium and are facing the same stressor, JNK signalling is only induced in clusters of a certain size but not in surrounding cells. Increased JNK activation was also observed in the p38α deleted intestinal epithelium of a mouse model for IBDs [53]. Furthermore, ulcerations occur in similar patchy patterns in IBDs [54]. Thus, the *MK2* mutant phenotype may be useful to decipher how a group of cells within a tissue of genetically identical cells transforms into the weakest link in the chain upon stressful conditions.

Several lines of evidence support the notion that the p38b/MK2 signalling complex is key to EC protection against chronic salt stress. (1) *p38b* and *MK2* mutant larvae both develop BDs upon stress conditions. (2) The severity of the *p38b*; *MK2* double mutant phenotype upon stress is similar to the *p38b* single mutant phenotype, suggesting that they act in the same signalling pathway. (3) p38b but not p38a physically associates with MK2 via its C-terminal CD domain (DPTD motif). (4) The binding of p38b to MK2 stabilises MK2. (5) Upon co-expression, p38b but not p38a redirects MK2 to the cytoplasm. (6) Both the activation and the catalytic activity of p38b are required to efficiently relocalise MK2. (7) The binding of MK2 to p38 and the catalytic activities of both kinases are essential to protect the ECs of the larval hindgut upon salt stress. Taken together, stabilisation, localisation and activation of MK2 by p38b are required for a proper stress response.

Our genetic analysis also revealed that p38 SAPK signalling is contributing to stress protection in different ways in addition to the pivotal role of the p38b/MK2 complex. First, p38b impacts on hindgut homeostasis in an MK2-independent manner. This is apparent from the *p38b* mutant larvae that, in contrast to *MK2* mutant larvae, develop BDs even at normal conditions. Consistently, a p38b protein version that no longer binds MK2 partially rescues the *p38b* mutant phenotype. Second, p38a is also required for full stress protection. The strong phenotype of *MK2; p38a* double mutants underscores the importance of the p38a SAPK pathway upon severe salt stress. However, the double mutants do not display an increase in BD formation but rather a decrease in viability. Thus, p38a may be involved in general stress protection that is not specific to the hindgut tissue. Third, a common p38 SAPK branch, encompassing p38a, p38b and potentially also p38c, is essential as the *p38a; p38b* double mutants die. Since the *p38a^1^* allele affects the coding sequences of *p38a* and *p38c*, the *p38a^1^; p38b^d27^* double mutants may even represent *p38a; p38b; p38c* triple mutants. However, it is unclear to date whether *p38c* is a pseudogene. Recent studies have suggested an involvement of p38c function in immune gene regulation, early larval survival, and fertility [41], [55]. Thus, further studies will be required to clarify whether p38c contributes to p38 signalling subbranches. Finally, the slight increase in BDs seen in *MK2; p38b* double mutants under normal conditions suggests that MK2 also performs a p38b-independent function in the hindgut. *p38b* mutants always impact on MK2 signalling since the MK2 protein is not stable and probably not correctly localised if not bound to p38b. Negative feedback regulation acting from MK2 on the activation of p38b further complicates the SAPK signalling network.

What are the upstream components regulating the p38b/MK2 complex? To our surprise, MKK3/Lic does not appear to play a role in the hindgut function of the p38b/MK2 branch. MKK3 but not MKK4 can activate p38 proteins in cell culture [56]. On the other hand, siRNA mediated knockdown of both *MKK4* and *MKK3* is required to fully block the activation of p38 under certain stresses in S2 cells [57]. Both *p38b* and *MKK3/lic* mutants show a strong reduction in p38 activation but no BDs are observed in *MKK3/lic* mutants. In contrast, mutants for *MEKK1*, which acts upstream of *MKK3*, do develop BDs similar to *p38b* and *MK2* mutants. In mammalian cells, it has been shown that p38 can be activated independently of a MAP2 kinase [58]–[60]. However, no activation of p38 occurred in fibroblasts of *MKK3 MKK6* double mutant mice [61]. Since MKK4 is a suppressor of the BD phenotype and MKK7 most likely does not activate p38 in *Drosophila*, a scenario of MAP2K-independent p38 activation could also apply for the p38b/MK2 signalling branch in the larval hindgut (Figure 7).

Interestingly, overexpression of the kinase-dead or of the non-activatable p38b protein version results in an even stronger phenotype than the deletion of p38b, probably by titrating upstream partners that would have additional functions besides activating p38b. A common p38a/p38b activator would be a strong candidate for such an upstream component. Saturating this common p38 activator with p38b^KR^ or p38b^AGF^ would essentially result in a *p38a/p38b* double mutant situation and therefore would explain the strong phenotype, especially at high salt conditions.

Our analysis of the p38b/MK2 signalling module in hindgut ECs reveals how deletion of SAPK members results in increased sensitivity towards a particular stressor from the molecular level to the level of the whole organism. These findings provide a new model of how hindgut homeostasis is maintained and how different SAPK branches act together *in vivo* to ensure cellular survival upon stress. The p38 SAPK pathway efficiently protects hindgut ECs over a wide range of stress conditions and for an astonishingly long time period of at least five days without cell replenishment. In this light, a comparative analysis of the larval and the adult hindgut (which has to be maintained for a time period of up to forty days) will be of great interest.

How does p38 interfere with JNK activation in the hindgut epithelium? In mammalian cells, p38α binds to and phosphorylates TAB1 (and potentially TAB2). As a consequence, the activities of TAK1 and thereby JNK are reduced [62]. TAB1 is not conserved in *Drosophila* but TAB2 might be involved in a similar negative feedback loop. TAB2 has been implicated in JNK activation in response to peptidoglycans and lipopolysaccharides. However, in the absence of TAB2, no change in JNK activation in response to Sorbitol or NaCl has been observed [63], [64]. Alternatively, p38 might induce a JNK phosphatase. p38α has been shown to impact on JNK activation by inducing DUSP1/MKP-1 in mammalian cells. Sustained JNK activation due to a loss of DUSP1/MKP-1 resulted in increased cell death in response to UV stress [65]. Interestingly, p38 activation was also increased in DUSP1/MKP-1 mutant cells but p38 activity was not linked to cell death induction. Similarly, *MK2* mutant larvae bearing BDs display an overactivation of both p38 and JNK, and blocking JNK only is sufficient to prevent the BD phenotype.

Despite the more complex nature of mammalian guts, the strong conservation of stress-signalling pathways and the similar demands of ECs make it likely that our results will also be important in the context of various diseases of the intestinal system. A variety of different signalling pathways have been implicated in IBDs, underscoring the complex nature of these diseases. p38 and MK2 are critical regulators of TNFα production and are thereby associated with IBDs [8] but the role of p38 SAPK in IBDs has remained controversial [9], [10]. Our study identifies a crucial role of p38b/MK2 signalling in the first line of defence against a particular stressor in a model system devoid of an adaptive immune system. The consequences of lacking this immune system-independent protective function of a SAPK branch might parallel early steps of IBD development in intestinal epithelial cells. Indeed, a recent study has revealed tissue-specific effects of p38α in a DSS-induced mouse model for IBDs [53]. Deleting p38α in the myeloid lineage had beneficial effects, consistent with the inflammatory nature of IBDs. In contrast, p38α deletion in the intestinal epithelium resulted in a loss of gut homeostasis, marked by increased proliferation and by a reduction in goblet cells. Thus, our studies on *Drosophila* p38 signalling and its role in the larval hindgut provide a basis to specifically address the role of ECs in the maintenance of an intestinal epithelium in the absence of proliferation and immune response.

## Materials and Methods

### Fly Media and Stock Keeping

1 litre *Drosophila* media contains 100 g fresh yeast, 55 g cornmeal, 10 g wheat flour, 75 g sugar, and 8 g bacto-agar. For stress medium, *Drosophila* media were boiled and sodium chloride was added from a 5 M stock solution. 15 ml/l of a stock solution containing 33 g/L nipagin and 66 g/L nipasol in 96% EtOH was added to prevent growth of mould and bacteria. All crosses and experiments were performed at 25°C.

### Fly Stocks

*GE3296* was remobilised to generate the *MK2* deletion mutants. *y w; MK2* (genomic rescue), *y w; MK2-GFP* (genomic GFP reporter); *y w; 86Fb pattB [GST-X] (X…p38a, p38b, p38a>b, p38b>a, p38b^KR^, p38b^AGF^), y w; 51D [MK2], y w; 51D [MK2^KD^], y w; 51D [mouseMK2], y w; nbyn2-GAL4* were generated in this study. For overexpression analyses, the following lines were used: *y w; cad-GAL4/CyO y+, y w; byn-GAL4/TM6B, y w; en-GAL4/CyO y+, y w; NP1-GAL4/CyO y+, y w; da-GAL4* and *y w; Act-GAL4*. For genetic interactions, the following lines were used: *y w; FRT82B D-p38a^1^/TM6B* [66], *y w; p38b^d27^/CyO y+* [46], *y w licorne^d13^/Binsn* [46], *y w; MKK4^414^/TM6B, y w; MKK4^589^/TM6B* [67]; *w dTAK1^2^*, *w dTAK1^4^* [63], *y w; MEKK1^Ur36^/TM6B* [45]. For hemocyte ablation, *y w; He-GAL4, UAS-GFP* flies were crossed to *y w; UAS-Bax* flies [68]. The following reporter lines have been used: *nrg^G00305^* [69], *y w; cecA1-lacZ* [70]; *y w; puc^E69^*, *y w; UAS-EGFP; puc-GAL4/TM6B* (gift from K. Basler), and *y w; msn-lacZ* [52].

### Stress Experiments

Females were allowed to lay eggs overnight on apple agar plates. Eggs were collected and 80–120 eggs were transferred to the different food vials. For BD quantification, larvae were analysed before reaching L3 wandering stage. For survival quantification, dead embryos were counted 24 h after seeding to the food and survival to pupae was recorded, respectively.

### Plasmid Constructs and Transgenic Animals

For the *MK2* genomic rescue construct, the genomic region between *CG15771* and *CG15770* was PCR-amplified and cloned into *pCasper3*. For the genomic *MK2*-*GFP* reporter, the same region was used but the *MK2* coding sequence was replaced by the *GFP* coding sequence.

For overexpression constructs, the *MK2* coding sequence was amplified and cloned into *pENTR/D-TOPO* (Invitrogen). For the kinase-dead MK2 protein, the mutation leading to the K49A substitution was introduced by PCR mutagenesis. The inserts were shuttled into the destination vector pTGW (http://www.ciwemb.edu/labs/murphy/Gateway%20vectors.html) for N-terminal GFP tagging. To express untagged MK2, the *MK2* coding sequence was cut from the *pENTR/D-TOPO* and ligated into a *pUAST-attB* vector.

GST-tagged p38a and p38b overexpression constructs were generated by ligating the GST coding sequence in frame to the p38a or p38b coding sequence, and the resulting fusion sequences were cloned into a *pUAST-attB* vector. The constructs encoding the p38a or p38b protein mutants were generated by PCR mutagenesis. In the p38a→b and the p38b→a protein mutants, the EPSV motif was changed to DPTD and vice versa. The kinase-dead or non-activatable p38b protein mutants were generated by introducing mutations in the coding sequence that result in the K53R substitution and in the replacement of the TGY dual phosphorylation motif by AGF, respectively.

*pUAST-attB* based constructs were injected into embryos carrying a landing site (*vas-ϕC31-zh2A; ZH-attP-51D* for chromosome II and *vas-ϕC31-zh2A; ZH-attP-86Fb* for chromosome III, [71]). The *MK2* genomic rescue and reporter lines were generated by co-injecting the respective plasmid with *Δ2–3* helper plasmid into *y w* embryos.

### Cell Transfection and Pull Downs

Transfection of S2 cells was done using the Effectene Transfection Reagent (Qiagen) according to the manufacturer's protocol. After four days of protein expression, cells were harvested and lysed. Pull down of GST-p38 proteins was performed using glutathione sepharose beads (Pharmacia Biotech AB). 10% of the lysates was loaded as input and the complete pull down sample was loaded onto SDS PAGE.

### Dissection and Fixation of Larval Tissues

Larvae were collected in PBS on ice. Three to five larvae were transferred to new vials containing 400 µl ice-cold PBS. Larvae were dissected and the desired organs were transferred into a microfuge tube containing 500 µl of ice-cold 4% paraformaldehyde in PBS. Hindguts and midguts were fixed for 40–50′ at 4°C. Subsequently, the fixative was removed by three washing steps with cold PBS. Fixed preparations were stored in PBS at 4°C until furthre use.

### Preparation of Cells for IHC

Cover slips were washed by dipping into 100% EtOH and air-dried. They were then incubated in 0.15% ConA solution (in ddH_2_O) for 1–2 h, washed with ddH_2_O and air-dried overnight.

ConA slides were placed into a small Petri dish and covered with Schneider's medium (approx. 1 ml). 200 µl S2 cells were added and allowed to settle onto the discs for 45′ to max. 90′. Cover slips were then washed once with ice-cold PBS, and 1 ml 4%PFA was added for 5′ fixation on ice followed by 10′ fixation at room temperature. Cover slips were washed three times with PBS. Cells were then permeabilised with PBT for 10′ and stored in PBS until further processing.

### Antibody Stainings

Specimens were blocked by incubating in 2% NDS in PBS with 0.2% Triton X-100 (or 0.3% Triton X-100 for hindguts) for 1 h (larval tissues) or 30′ (S2 cells), respectively. Primary antibodies were added in PBS with 2% NDS and 0.2% Triton X-100 for 1 h (S2 cells) or overnight (larval tissues), respectively. Before secondary antibodies were added, samples were washed three times in PBS with 0.2% Triton X-100. Secondary antibodies were added in PBS with 2% NDS and 0.2% Triton X-100 for 1 h (S2 cells and larval tissues).

Western blot (WB) membranes were blocked in 3% membrane blocking agent (GE Healthcare) for one hour. Membranes were incubated with the primary antibodies overnight and one hour with the secondary antibodies in 3% membrane blocking agent.

*Primary antibodies*: rabbit anti-GST (1∶5,000 WB or 1∶500 IHC, Sigma G7781); rabbit anti-activated JNK (1∶1000 WB, Promega V793A); rabbit anti-pTGpY-p38 (1∶1000 WB, Cell Signaling 4631); mouse anti-Tubulin (1∶10,000 WB, Sigma T9026); rabbit anti-D-p38b (1∶1000 WB, [72]), and mouse anti-Hemese (1∶50, [73], [74]). The anti-*Drosophila* MK2 antibody was generated by Eurogentec by immunising a rabbit with the peptide H_2_0-QPKTTPLTDDYVTSN-COOH, and the final bleed was used 1∶500 in WB.

*Secondary antibodies:* HRP-coupled anti-mouse IgG (Jackson ImmunoResearch; 1∶10,000; WB), HRP-coupled anti-rabbit IgG (Jackson ImmunoResearch; 1∶10,000; WB), and Cy3-coupled anti-rabbit IgG (1∶500; IHC).

### Other Histological Stainings

Larvae were dissected and fixed by standard procedures. After washing with PBS, 500 µl X-gal staining solution was added, and the samples were incubated at 37°C in the dark. The staining progress was observed every 10′, and the staining reaction was stopped by two washes with PBT.

Alexa Fluor 594-conjugated phalloidin (Molecular Probes) was used to stain muscles. For apoptosis detection, the TUNEL assay kit ApopTag RED In Situ Detection Kit (Millipore S7165) was used.

### Hindgut Sections

Larvae were dissected on ice and hindguts were immediately fixed in 2.5% glutaraldehyde, 1% paraformaldehyde, 1% potassium ferrocyanide, 0.1 M cacodylate buffer for 80′. After washing three times in 0.1 M cacodylate buffer, hindguts were postfixed in 1% osmium tetroxide, 1% potassium ferrocyanide, 0.1 M cacodylate buffer (pH 7.4) for 60′. Hindguts were then dehydrated in an ascending acetone series (30%>50%>70%>90%>100% 3′ each and 5′ 100%). The samples were incubated overnight in a 1∶1 acetone∶Spurr solution. After equilibration in Spurr solution for 4 h, samples were embedded in Spurr solution and hardened at 65°C overnight. 2 µm sections were made with a Supercut Reichert-Jung 2050 microtome, and sections were mounted in DPX Mountant for histology (Fluka).

## Supporting Information

Figure S1 *MK2* null mutants and development of BDs. (A) Larval extracts of wild-type (*Δ1A*) larvae but not of *MK2* mutants (*Δ43*) have *in vitro* kinase activity towards small heat shock protein 25 (hsp25). Overexpression of wild-type MK2, but not of kinase-dead MK2, boosts hsp25 phosphorylation. Antibodies against *Drosophila* MK2 do not recognize a band in Western analysis on *MK2* mutant total larval lysates. (B) *MK2* mutants were tested for the appearance of BDs by feeding different stressors. A rough classification reveals that only high salt and SDS feeding induce a BD phenotype. (C) Representative pictures of *MK2* mutant (*Δ43*) larvae at indicated time points (white arrows point to BDs in the first two panels). (D) Quantification of BDs of *MK2* mutant larvae reared on 0.2 M NaCl food at the time points depicted in (C). (E) The size of the BDs depends on the strength of the stress. *MK2* mutant larvae were reared on 0.1 M or 0.2 M NaCl food, respectively, and BDs were analysed in L3 before wandering stage. White arrows point to BDs in the first two panels.
(TIF)

Figure S2 Hindgut defects and hindgut-specific rescue of *MK2* mutants. (A) Hemocytes are observed neither in wild-type (*Δ1A*) nor in *MK2* mutant (*Δ43*) hindguts. No Hemese staining is found at the melanised lesion site (lowest panel) and in *MK2* mutant hindguts without BDs (middle panel), even when the visceral musculature (red) is damaged (lowest panel). Staining of blood cells attached to the cuticle (inset and white arrow in the middle panel) demonstrates that the staining protocol worked. (B) Hemocytes are dispensable for BD formation as larvae lacking hemocytes still develop BDs (white arrow). (C) Bright field pictures of hindgut sections of wild-type (*Δ1A*) and *MK2* mutant (*Δ43*) larvae reared on 0.1 M NaCl. In *MK2* mutants without BDs, the hindgut structure appears either undamaged (second panel) or displays blistering of ECs in the dorsal hd domain (black asterisk in third panel). EC blistering is also observed in *MK2* mutants with BDs at a distance of the BD (black asterisk in fourth panel). Panels four and five show sections of the same hindgut ahead of and at the BD lesion site, respectively. White asterisks mark the gut content; black arrows point to undamaged apical membranes; white arrow indicates BD. (D) Various GAL4 lines were used to drive MK2 expression from a wild-type *UAS-MK2* cDNA construct in an *MK2* mutant background (*Δ43*), and the ability to rescue the BD phenotype was scored. Only ubiquitous and hindgut-specific expression of catalytically active but not of a kinase-dead MK2 rescues the BD phenotype.
(TIF)

Figure S3 *p38b* BD phenotype rescued by p38 expression. Homozygous *p38b* mutants were reared on 0.2 M NaCl food. Rescue of the BD phenotype by *p38a* and *p38b* was quantified (Figure 4C). Here we show representative examples of larvae that were quantified for their BD appearance.
(TIF)

Figure S4 Systemic effects observed in *MK2* mutants. (A) *MK2* mutants reared on 0.2 M NaCl food often display a severely ruptured hindgut musculature (white arrows), resulting in a local gut barrier breakdown. The BD can be recognized based on its autofluorescence (yellow). (B) In such strongly affected larvae, the antimicrobial peptide CecA1 is induced in the midgut, indicative of a systemic response. The black bar (labelled with hg) indicates the hindgut; the black arrow marks the BD; the white arrows point to the CecA1-lacZ induction (blue). (C) The systemic disturbance in larvae with large BDs (asterisk) is underscored by the appearance of melanised pericardial cells (arrow).
(TIF)
