# Supplementary figures and images for: MK2-Dependent p38b Signalling Protects Drosophila Hindgut Enterocytes against JNK-Induced Apoptosis under Chronic Stress
Source: PLoS Genet. 2011 Aug 4;7(8):e1002168. doi: 10.1371/journal.pgen.1002168 (PMC3150449; doi:10.1371/journal.pgen.1002168)

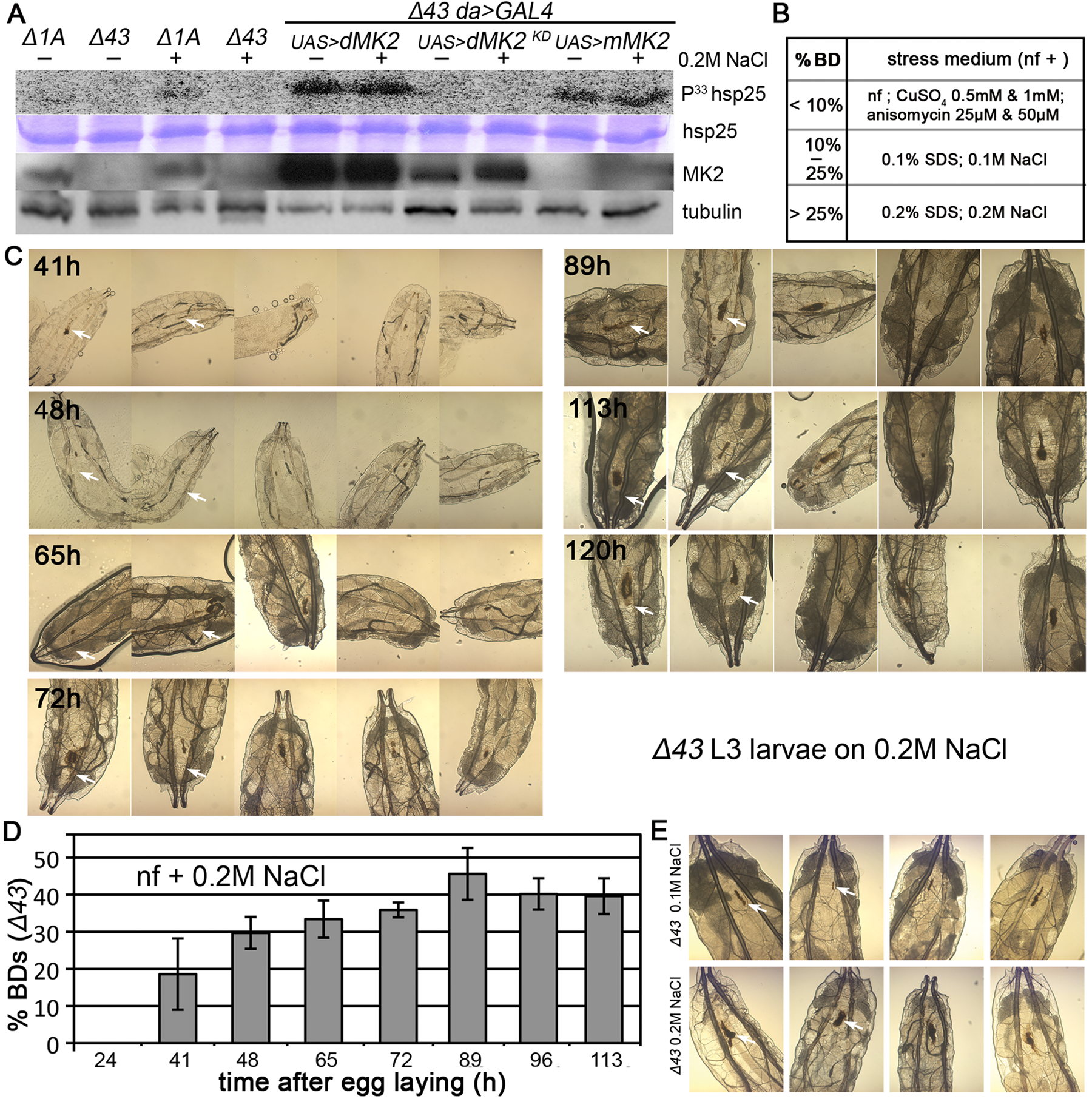

Supplement: Figure S1 — MK2 null mutants and development of BDs. (A) Larval extracts of wild-type (Δ1A) larvae but not of MK2 mutants (Δ43) have in vitro kinase activity towards small heat shock protein 25 (hsp25). Overexpression of wild-type MK2, but not of kinase-dead MK2, boosts hsp25 phosphorylation. Antibodies against Drosophila MK2 do not recognize a band in Western analysis on MK2 mutant total larval lysates. (B) MK2 mutants were tested for the appearance of BDs by feeding different stressors. A rough classification reveals that only high salt and SDS feeding induce a BD phenotype. (C) Representative pictures of MK2 mutant (Δ43) larvae at indicated time points (white arrows point to BDs in the first two panels). (D) Quantification of BDs of MK2 mutant larvae reared on 0.2 M NaCl food at the time points depicted in (C). (E) The size of the BDs depends on the strength of the stress. MK2 mutant larvae were reared on 0.1 M or 0.2 M NaCl food, respectively, and BDs were analysed in L3 before wandering stage. White arrows point to BDs in the first two panels. (TIF) [file pgen.1002168.s001.tif]

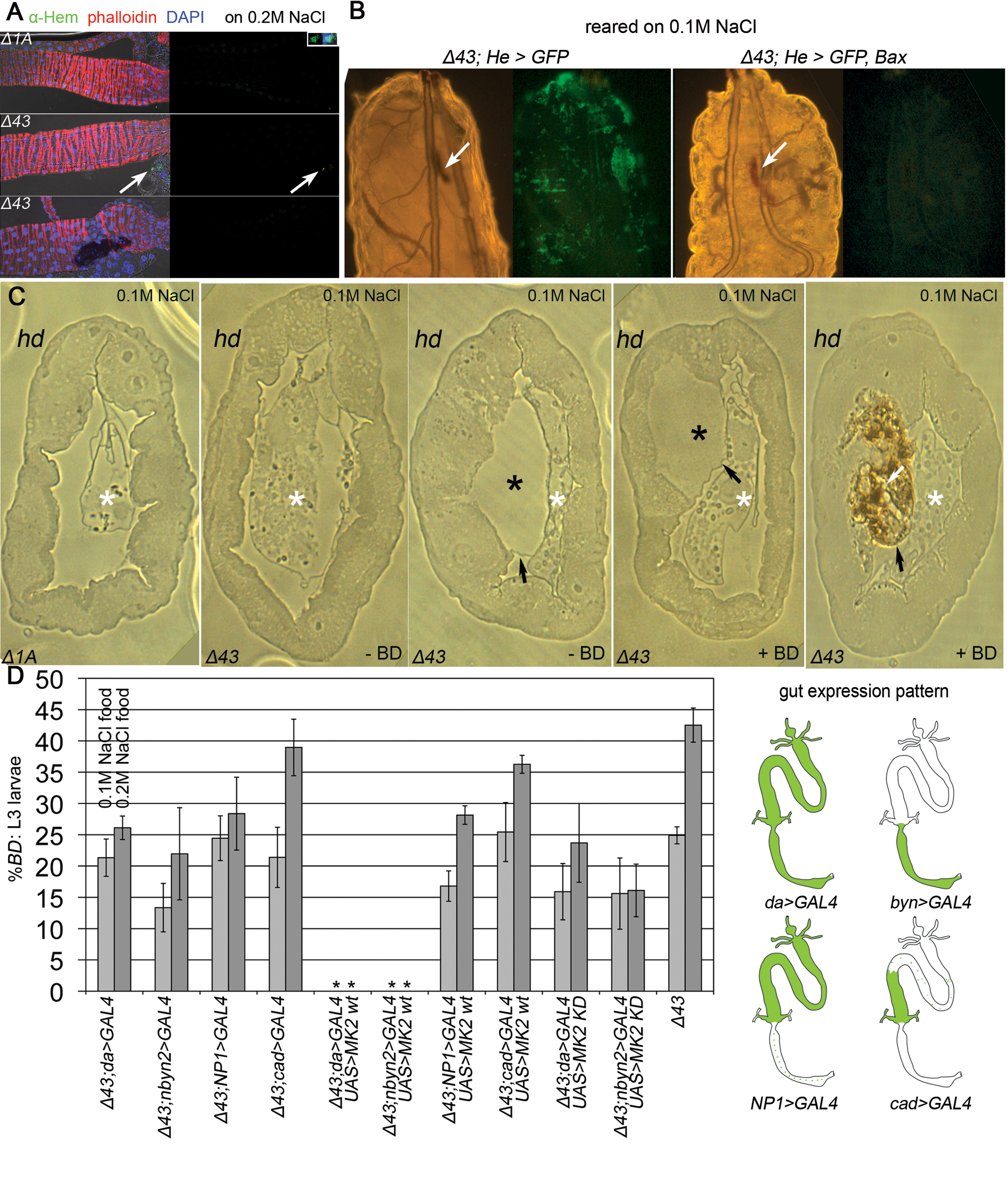

Supplement: Figure S2 — Hindgut defects and hindgut-specific rescue of MK2 mutants. (A) Hemocytes are observed neither in wild-type (Δ1A) nor in MK2 mutant (Δ43) hindguts. No Hemese staining is found at the melanised lesion site (lowest panel) and in MK2 mutant hindguts without BDs (middle panel), even when the visceral musculature (red) is damaged (lowest panel). Staining of blood cells attached to the cuticle (inset and white arrow in the middle panel) demonstrates that the staining protocol worked. (B) Hemocytes are dispensable for BD formation as larvae lacking hemocytes still develop BDs (white arrow). (C) Bright field pictures of hindgut sections of wild-type (Δ1A) and MK2 mutant (Δ43) larvae reared on 0.1 M NaCl. In MK2 mutants without BDs, the hindgut structure appears either undamaged (second panel) or displays blistering of ECs in the dorsal hd domain (black asterisk in third panel). EC blistering is also observed in MK2 mutants with BDs at a distance of the BD (black asterisk in fourth panel). Panels four and five show sections of the same hindgut ahead of and at the BD lesion site, respectively. White asterisks mark the gut content; black arrows point to undamaged apical membranes; white arrow indicates BD. (D) Various GAL4 lines were used to drive MK2 expression from a wild-type UAS-MK2 cDNA construct in an MK2 mutant background (Δ43), and the ability to rescue the BD phenotype was scored. Only ubiquitous and hindgut-specific expression of catalytically active but not of a kinase-dead MK2 rescues the BD phenotype. (TIF) [file pgen.1002168.s002.tif]

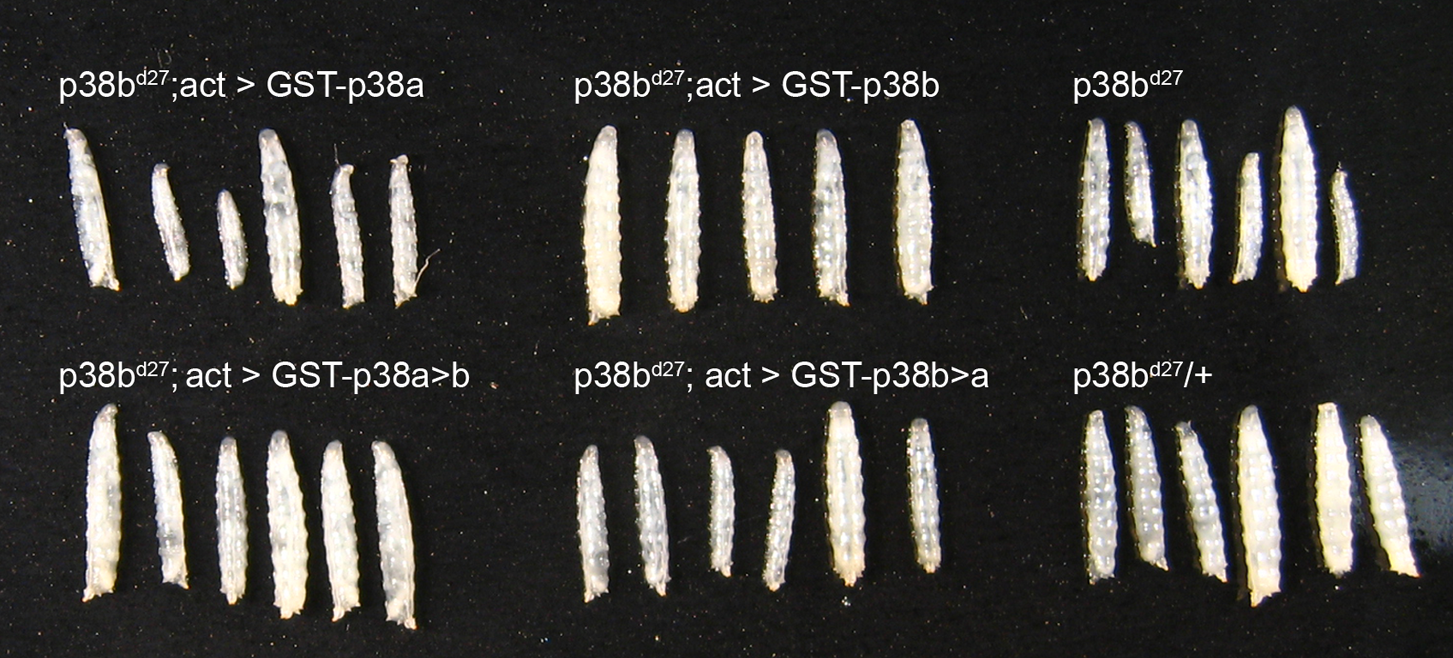

Supplement: Figure S3 — p38b BD phenotype rescued by p38 expression. Homozygous p38b mutants were reared on 0.2 M NaCl food. Rescue of the BD phenotype by p38a and p38b was quantified (Figure 4C). Here we show representative examples of larvae that were quantified for their BD appearance. (TIF) [file pgen.1002168.s003.tif]

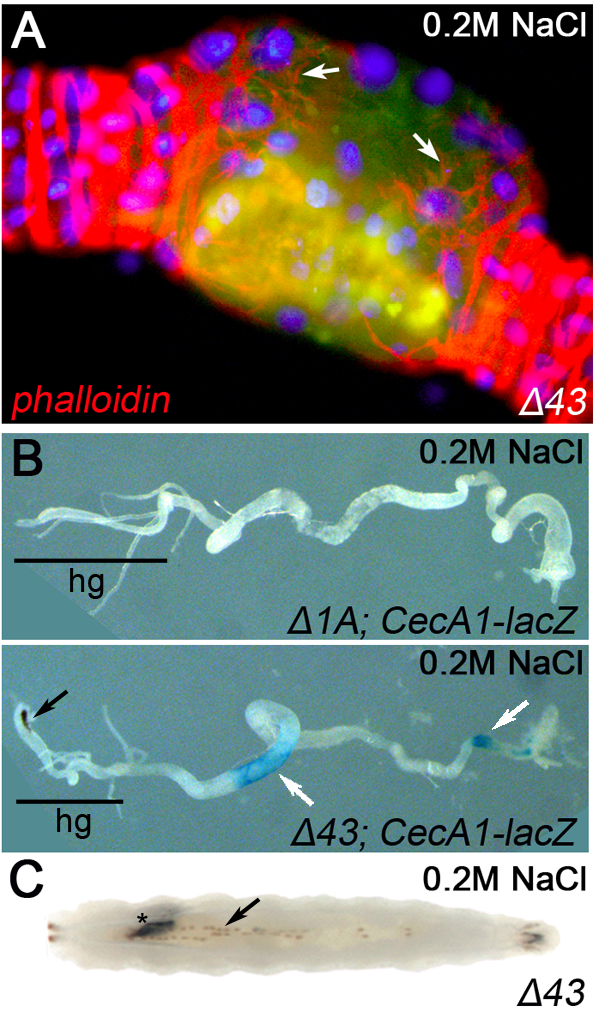

Supplement: Figure S4 — Systemic effects observed in MK2 mutants. (A) MK2 mutants reared on 0.2 M NaCl food often display a severely ruptured hindgut musculature (white arrows), resulting in a local gut barrier breakdown. The BD can be recognized based on its autofluorescence (yellow). (B) In such strongly affected larvae, the antimicrobial peptide CecA1 is induced in the midgut, indicative of a systemic response. The black bar (labelled with hg) indicates the hindgut; the black arrow marks the BD; the white arrows point to the CecA1-lacZ induction (blue). (C) The systemic disturbance in larvae with large BDs (asterisk) is underscored by the appearance of melanised pericardial cells (arrow). (TIF) [file pgen.1002168.s004.tif]
